# Insulin-like growth factor binding protein 5 enhances survival of LX2 human hepatic stellate cells

**DOI:** 10.1186/1755-1536-3-3

**Published:** 2010-02-17

**Authors:** Aleksandar Sokolović, Milka Sokolović, Willem Boers, Ronald PJ Oude Elferink, Piter J Bosma

**Affiliations:** 1 Tytgat Institute for Liver and Intestinal Research, Academic Medical Center, University of Amsterdam, Amsterdam, The Netherlands; 2 Department of Medical Biochemistry, Academic Medical Centre, University of Amsterdam, Amsterdam, The Netherlands

## Abstract

**Background:**

Expression of insulin-like growth factor binding protein 5 (IGFBP5) is strongly induced upon activation of hepatic stellate cells and their transdifferentiation into myofibroblasts *in vitro*. This was confirmed *in vivo *in an animal model of liver fibrosis. Since IGFBP5 has been shown to promote fibrosis in other tissues, the aim of this study was to investigate its role in the progression of liver fibrosis.

**Methods:**

The effect of IGFBP5 was studied in LX2 cells, a model for partially activated hepatic stellate cells, and in human primary liver myofibroblasts. IGFBP5 signalling was modulated by the addition of recombinant protein, by lentiviral overexpression, and by siRNA mediated silencing. Furthermore, the addition of IGF1 and silencing of the IGF1R was used to investigate the role of the IGF-axis in IGFBP5 mediated effects.

**Results:**

IGFBP5 enhanced the survival of LX2 cells and myofibroblasts via a >50% suppression of apoptosis. This effect of IGFBP5 was not modulated by the addition of IGF1, nor by silencing of the IGF1R. Additionally, IGFBP5 was able to enhance the expression of established pro-fibrotic markers, such as *collagen Iα1*, *TIMP1 *and *MMP1*.

**Conclusion:**

IGFBP5 enhances the survival of (partially) activated hepatic stellate cells and myofibroblasts by lowering apoptosis via an IGF1-independent mechanism, and enhances the expression of profibrotic genes. Its lowered expression may, therefore, reduce the progression of liver fibrosis.

## Background

The extensive accumulation of extracellular matrix (ECM) produced by activated hepatic stellate cells (HSC), which normally reside in the space of Disse as the major vitamin A storage site, is a hallmark of liver fibrosis [1,2]. Liver damage induces HSC activation and, upon repeated and/or chronic injury, they transdifferentiate into myofibroblast-like cells [1,3]. These cells migrate to the damaged regions of the liver [4-6] where they play a central role in the pathogenic deposition of ECM [7,8].

In order to identify novel therapeutic targets, we used gene expression profiling at different stages of the pathogenic transdifferentiation of HSC [9]. One of the factors found to be upregulated upon HSC activation and further enhanced upon transdifferentiation into myofibroblasts was IGFBP5 (insulin-like growth factor binding protein 5). This strong induction of IGFBP5 expression was confirmed during the development of liver fibrosis in the Mdr2^-/- ^mice, a well established animal model of liver fibrosis [10]. Expression of IGFBP5 in HSC has been reported to be enhanced by insulin-like growth factor 1 (IGF1) via a post-translational mechanism, while its novel synthesis was decreased by TGFβ1 (transforming growth factor beta 1) [11].

IGFBP5 is a member the IGFBPs that bind IGF1 [12-14]. IGF1 is mainly synthesized by the liver and gets secreted into the circulation bound to IGFBPs, which prolong its half-life and, by modulating its interaction with the IGF1 receptor (IGF1R), control its biological activity [12,15,16]. In advanced liver fibrosis, the IGF1 axis is severely impaired mostly due to a reduced number of healthy IGF1 producing hepatocytes [17]. The decrease in IGF1 signalling seems to provide a pro-fibrotic environment, since the progression of liver fibrosis could be delayed by IGF1 administration [18,19]. As IGFBP5 impairs the binding of IGF1 to the cell-surface receptor IGF1R [20], its increased expression in activated HSC and myofibroblasts may reduce IGF1 signalling and, thus, promote liver fibrosis. In contrast, in another study IGF1 has been reported to exert pro-fibrotic activity [21]. In that case the inhibition of IGF1 signalling by IGFBP5 would impair the pathogenesis of liver fibrosis.

In lung and skin, IGFBP5 has also been shown to induce fibrosis upon epithelial injury [13,22,23]. Induction of IGFBP5 expression initiated the activation and transdifferentiation of resident fibroblasts into myofibroblasts, causing increased ECM production and deposition in these tissues. Moreover, it seemed to cause cellular senescence and epithelial-mesenchymal transition [24].

The aim of this study was to investigate the role of IGFBP5 in liver fibrosis by employing both gain and loss of function approaches. We focused on the effect of IGFBP5 on HSC, using the human LX2 cell line [25], which recapitulates many features of the activated HSC phenotype. In addition, to see if IGFBP5 could play a role in more advanced stages of fibrosis, we analysed its effect on human primary liver myofibroblasts.

## Materials and methods

### Cell culturing

LX2 cells (kindly provided by Prof. dr. S. Friedman) and human myofibroblasts (obtained as described [9]) were cultured in Dulbecco's modified Eagle's medium (Lonza, Verviers, Belgium), supplemented with 10% fetal calf serum (FCS), 1 mmol/l L-glutamine, 100 IU/ml penicillin and streptomycin. Human recombinant IGFBP5 and IGF1 (rIGFBP5 and rIGF1; Gro-Pep, Reutlingen, Germany) were added in concentration 0.1 ng/μl [26] and 1 ng/μl, respectively, at 24 and 45 h of cell culturing. The cells were used for additional assays 3 h after the last protein additions.

In order to induce apoptosis, the cells were serum deprived for 48 h. For drug induced apoptosis, the cells grown in 10% FCS were incubated with 0.5 μM gliotoxin (Alexis Biochemicals, Lausen, Switzerland) for 3 h. This dose induced caspase activity sixfold 3 h after gliotoxin administration (data not shown).

### Lentiviral transduction

A lentiviral vector encoding human IGFBP5 behind the constitutive PGK promoter was generated using the self-inactivating cppt-PGKiresGFP-PRE vector [27]. Sequencing was performed to exclude mutations.

### siRNA mediated silencing

IGFBP5, IGF1R and negative control small interfering RNAs (siRNAs) were purchased from Invitrogen (Breda, Netherlands). siRNA was introduced to the cells using Lipofectamine 2000 (Invitrogen, Breda, Netherland) in Optimem (Gibco, Invitrogen, Paisley, UK), according to the manufacturer's instructions. For the downstream applications siRNA transfected cells were grown in 10% FCS and used 48 h after transfection. When apoptosis was induced by serum starvation, the cells were first grown o/n (16 h) in serum containing medium, which was then replaced for 48 h by serum depleted medium.

### Cell viability test

Cell Proliferation Reagent WST-1 (Roche Applied Science, Mannheim, Germany) was added (1/10th of the culture volume) to cells grown in 96-well plates. Absorbance was measured at 450 nm (reference at 630 nm) immediately and after 1 h of incubation at 37°C.

### Bromo-2'-deoxy-uridine (BrdU) assay

BrdU assay (Roche applied Science, Penzberg, Germany) was performed according to the manufacturer's protocol. The cells grown in 96 well plates were incubated with BrdU during the last 16 h of culturing.

### Apoptosis assay

The Apo-One homogeneous Caspase 3/7 assay (Promega, Medison, WI, USA) was performed according to the manufacturer's protocol. The Apo-One Caspase 3/7 reagent was added in a 1:1 ratio with medium and fluorescence was measured using Novostar plate reader at 521 nm.

### Reverse transcription and quantitative polymerase chain reaction (qPCR)

Total RNA was isolated using Trizol (Invitrogen, Breda, The Netherlands). RNA concentration was determined spectrophotometrically and the integrity was checked by gel electrophoreses. cDNA was synthesized using an oligo-dT primer and Superscript III (Invitrogen, Breda, The Netherlands). qPCR was performed on a LightCycler using FastStart DNA Master Plus SYBR Green I (Roche, Mannheim, Germany). The relative expression levels were calculated using the LinReg program [28] and for each sample normalized by the expression of housekeeping gene *36B4 *(acidic ribosomal phosphoprotein P0). Primer sequences are shown in Table 1.

**Table 1:** Primers sequences used in quantitative polymerase chain reaction.

Gene	Symbol	Primers (5'→3')
**Insulin-like growth factor binding protein 5**	*IGFBP5*	F: GTCACTCCCCAGAGAAGCTG
		R: CCCCTGCTTAGATTGCCATA

**Insulin-like growth factor 1 receptor**	*IGF1R*	F: GGTTGAGGTGAGAGGTTTGC
		R: CAAATTGGCCATGTTATTACCTT

**Acidic ribosomal phosphoprotein P0**	*36B4*	F: AGGCGTCCTCGTGGAAGTGA
		R: GCGGATCTGCTGCATCTGCT

**Matrix metallopeptidase 1**	*MMP1*	F: AGCTAGCTCAGGATGACATTGATG
		R: CTCCCCGAATCGTAGTTATAGCAT

**TIMP metallopeptidase inhibitor 1**	*TIMP1*	F: CACCCACAGACGGCCTTCT
		R: TCTGGTGTCCCCACGAACTT

**Collagen, type I, alpha 1**	*COL1A1*	F: GGCGGCCAGGGCTCCGAC
		R: AATTCCTGGTCTGGGGCACC

**B-cell CLL/lymphoma 2**	*BCL2*	F: GTTGGTGGGGTCATGTGTGTGGAGAG
		R: TAGCTGATTCGACGTTTTGCCTGA

### Western blot analysis

Cell extracts were analysed by Western blotting as described [29]. We used antisera against IGFBP5 (1:250; Abcam, Cambridge, UK), IGF1R (1:200; Santa Cruz Biotechnology, CA, USA), poly (ADP-ribose) polymerase (PARP; 1:100; Promega, Madison, USA) and β-actine (1:3000; Sigma, St Louis, USA). Goat anti-mouse (1:2500; Bio-Rad Laboratories, Veenendaal, The Netherlands) and goat anti-rabbit IgG horseradish peroxidase conjugated (1:5000; Santa Cruz Biotechnology, CA, USA) were used as secondary antibodies. Chemiluminescence was quantified on the Lumi-Imager F1 using CDP-Star (Roche, Mannheim, Germany). Amido-black staining and β-actine immuno-blot verified similar protein loading.

### Statistical analysis

All cell culturing experiments were performed *in quadruplo*, and were repeated at least three times. In order to assess the significance of the data, ANOVA analysis and Student's *t *test were employed. The error bars in the figures represent the standard deviation. Significance threshold was set to *P *< 0.05 (denoted by the asterisks in all the Figures).

## Results

### IGFBP5 expression in LX2 cells

In human HSC *in vitro*, IGFBP5 expression is enhanced twofold upon activation and 200-fold upon transdifferentiation into myofibroblasts [9]. LX2 cells also express IGFBP5 (Table 2). The 30% higher expression in these cells compared to resting HSC confirms reports demonstrating that it is a model for partially activated HSC [25].

**Table 2:** Relative gene expression of insulin-like growth factor 1 (IGF1) and insulin-like growth factor binding protein 5 (IGFBP5) in LX2 cells, activated hepatic stellate cells (HSC) and myofibroblasts given as fold changes of basal expression in freshly isolated human HSC.

	LX2 cells	HSC	Activated HSC	Myofibroblasts
IGFBP5	1.3	1	2.3	236

IGF1	0.7	1	1.2	0.3

**Figure 1:**
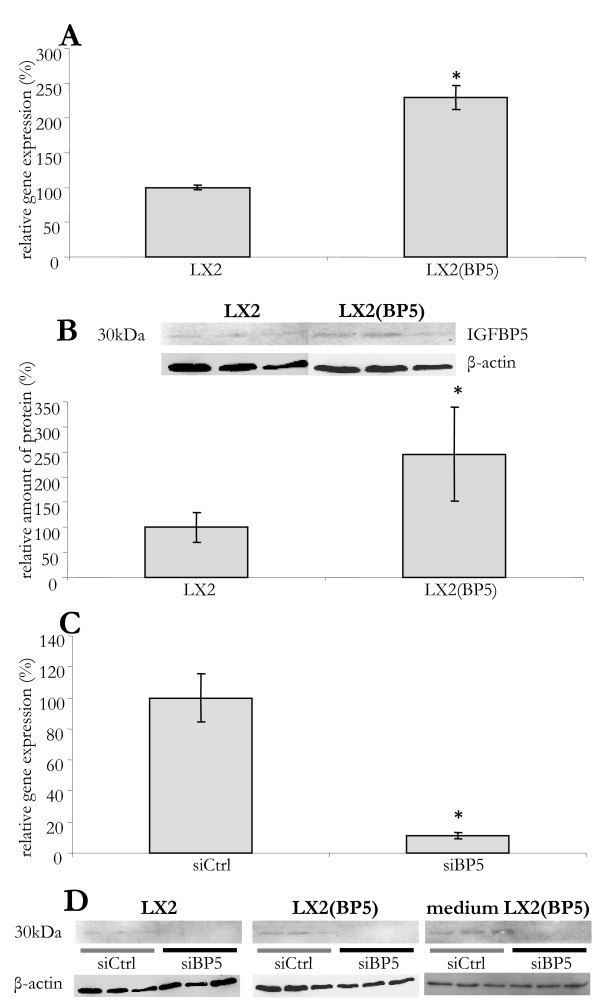
**Modulation of IGFBP5 expression in LX2 cells**. (A) *IGFBP5 *mRNA levels in control cells (LX2) and in cells with lentiviral overexpression of IGFBP5 (LX2(BP5)). RNA levels normalized to *36B4 *are shown as a percentage of control cells. (B) IGFBP5 protein levels in control (LX2) and transduced (LX2(BP5)) cells as demonstrated on a Western blot (upper panel) and quantified using the LumiImager (lower panel). (C) Silencing of *IGFBP5 *expression. Cells were transfected with small interfering (si) RNA for *IGFBP5 *(siBP5) or with a control siRNA (siCtrl). RNA was isolated 48 h later and IGFBP5 mRNA levels were quantified, normalized to *36B4 *and given as a percentage of control cells. (D) Proteins were isolated from the cells and the medium 48 h after small interfering RNA (sRNA) transfection. The presence of IGFBP5 protein was visualized by Western blotting in LX2 and LX2(BP5) cells transfected with control siRNA (siCtrl) and IGFBP5 siRNA (siBP5) and in medium obtained from transfected LX2(BP5). The results are given as a percentage of the control cells. The error bars represent standard deviations, with asterisks denoting significant difference (*P *< 0.05).

In order to investigate a potential role of IGFBP5 in liver fibrosis, we developed methods to modulate its expression in LX2 cells. The endogenous expression of *IGFBP5 *mRNA was doubled using lentiviral transduction (Figure 1A). This resulted in a 2.5-fold increase in protein levels (Figure 1B). Transfection with *IGFBP5 *siRNA reduced *IGFBP5 *mRNA 80% and led to complete absence of detectable IGFBP5 signal on a Western blot (Figure 1C and 1D, respectively).

### IGFBP5 enhances survival of LX2 cells

**Figure 2:**
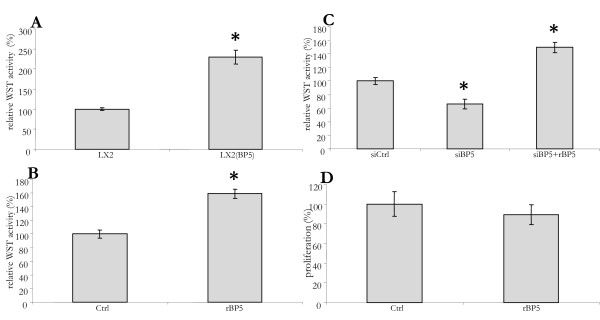
**IGFBP5 increases survival of LX2 cells**. (A) The viability of IGFBP5 overexpressing (LX2(BP5)) and control cells (LX2) cultured in serum-free medium for 48 h was measured using WST. (B) The viability of LX2 cells cultured in serum-free medium for 48 h was determined using WST. At *t *= 24 and 45 h, recombinant IGFBP5 was added (rBP5). (C) LX2 cells were transfected with small interfering control (siCtrl) or *IGFBP5 *siRNA (siBP5). At 16 h after transfection the medium was replaced by serum-free medium and 48 h later the viability of the cells was measured using WST. rIGFBP5 was added at *t *= 24 and 45 h of serum-free culturing (siBP5+rBP5). (D) Bromo-2'-deoxy-uridine (BrdU) incorporation was used to determine the proliferation of LX2 cells cultured in serum-free medium for 48 h. rIGFBP5 was added at *t *= 24 and 45 h (rBP5). BrdU was added at *t *= 32 h. The results are shown as percentage of the control cells.

Several studies have shown that IGFBP5 can affect cell survival [30-33]. We therefore compared the viability of control LX2 cells with cells over-expressing IGFBP5. When grown with 10% FCS, cell growth did not differ (data not shown). However, upon culturing in serum free medium for 48 h, a WST assay (for cell viability) indicated 100% higher viability of the transduced cells (Figure 2A). The similar 60% increase in viability seen upon addition of recombinant IGFBP5 (rIGFBP5) confirmed the effect of IGFBP5 on LX2 survival (Figure 2B). In serum free medium, the siRNA mediated reduction of *IGFBP5 *expression resulted in a >40% decrease in cell viability. This reduction in viability was prevented by the addition of rIGFBP5 to the silenced cells (Figure 2C).

In order to investigate if IGFBP5 affected proliferation, we studied its effect on the BrdU incorporation. In the cells grown in serum depleted medium the addition of 0.1 ng/μl rIGFBP5 at 24 h and 45 h of culturing did not effect the incorporation of BrdU (Figure 2D). This indicates that IGFBP5 does not enhance LX2 cell proliferation.

### Impact of IGFBP5 on apoptosis in LX2cells

**Figure 3:**
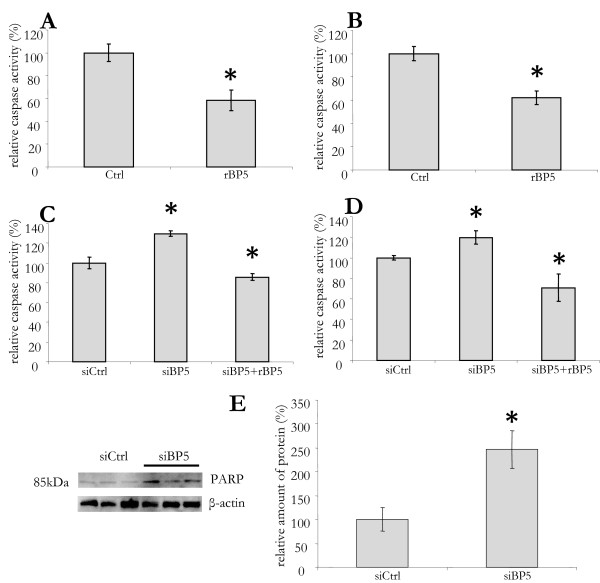
**IGFBP5 impact on apoptosis in LX2 cells**. (A) LX2 cells were cultured in serum-free medium for 48 h. Recombinant IGFBP5 (rBP5) was added at 24 h and 45 h and, at 48 h, a Caspase 3/7 assay was performed. (B) Cells grown in 10% fetal calf serum were treated with rBP5 as above, with the addition of 0.5 μM gliotoxin after 45 h. (C) LX2 cells were transfected with small interfering control (siCtrl) or *IGFBP5 *siRNA (siBP5). Sixteen hours after transfection, the medium was replaced by serum-free medium and cells were cultured for additional 48 h before performing the caspase assay. To *siIGFBP5 *transfected cells, recombinant IGFBP5 was added at *t *= 24 h and 45 h (siBP5+rBP5). (D) To cells treated with si- and r-BP5 as in C, 0.5 μM gliotoxin was added 3 h before performing the caspase assay. All results are shown as a percentage of the control cells. (E) Apoptosis was induced in LX2 cells transfected with control siRNA (siCtrl) or *IGFBP5 *siRNA (siBP5) by the addition of 0.5 uM gliotoxin at *t *= 45 h. Proteins were isolated at 48 h and Western blotting was performed for poly(ASP-ribose) polymerase detection (left panel). Quantification is shown in the right panel. β-actin was used as a loading control.

In order to investigate if the effect of IGFBP5 on viability was caused by decreased apoptosis, we determined the caspase activity using a Caspase 3/7 assay. The addition of rIGFBP5 to LX2 cells cultured in serum free medium did reduce the caspase activity by 40% (Figure 3A). In order to substantiate this protective effect, we also tested it in a model of drug induced apoptosis by gliotoxin administration [34]. The addition of rIGFBP5 3 h prior to the addition of 0.5 μM of gliotoxin resulted in a significant (40%) lowering of caspase activity (Figure 3B). In order to investigate if lowering of IGFBP5 would increase apoptosis, we studied the effect of siRNA mediated silencing on its expression. Compared to control-siRNA transfected cells, the caspase activity in silenced cells was increased by 30%. This increase was seen when apoptosis was induced both by serum starvation and gliotoxin. Administration of rIGFBP5 reduced caspase activity in silenced cells to a level significantly lover than that in control cells (Figure 3C and 3D, respectively). The increase in apoptosis in silenced cells was further confirmed by Western blot analysis, which demonstrated a 150% increase of poly (ADP-ribose) polymerase (PARP) protein expression in silenced compared to control cells (Figure 3E).

Altogether, these data indicate that IGFBP5 enhances the survival of this model for partially activated HSC by arresting apoptosis.

### IGFBP5 also enhances survival of human myofibroblasts

**Figure 4:**
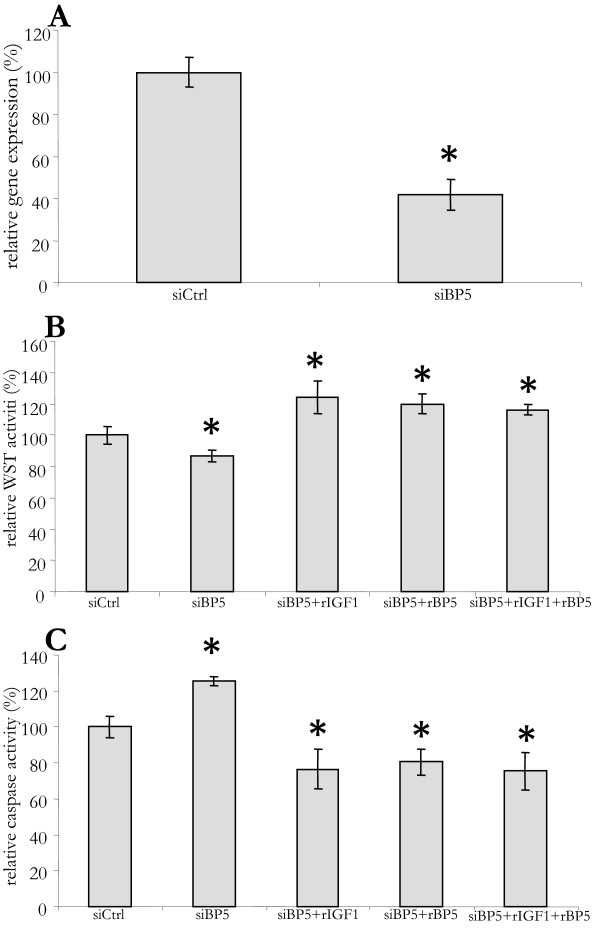
**In human myofibroblasts IGFBP5 silencing decreases survival and promotes apoptosis**. (A) Primary human myofibroblasts were transfected with small interfering (si) RNA for *IGFBP5 *(siBP5) or with a control siRNA (siCtrl). At 16 h after transfection the medium was replaced by serum-free medium. Cells were cultured for an additional 48 h. Then RNA was isolated and *IGFBP5 *mRNA level determined by quantitative polymerase chain reaction and normalized with *36B4*. (B) Recombinant IGFBP5 (rBP5), IGF1 (rIGF1), or both (rIGF1+ rBP5), where added at *t *= 24 h and 45 h, after replacing the medium, to siBP-5 transfected cells. At 48 h the WST assay was performed. (C) To cells treated as above but grown in 10% fetal calf serum, 0,5 μM gliotoxin was added at 45 h. Caspase 3/7 assay was performed at 48 h. Results are given as a percentage of control cells.

Activated HSC transdifferentiate into myofibroblasts in advanced stages of liver fibrosis. This transdifferentiation results in a further induction of *IGFBP5 *expression [9]. Given the effects on LX2 cells, IGFBP5 may also affect the survival of liver myofibroblasts. In order to investigate this, we performed siRNA mediated silencing of *IGFBP5 *expression in primary human myofibroblasts (Figure 4A). Forty-eight hours after transfection, *IGFBP5 *mRNA levels in primary myofibroblasts were decreased by 60%. Although the silencing was not as effective as that in LX2 cells, it did cause a small, but significant, decrease in survival, which could be prevented by the addition of both rIGF1 and rIGFBP5 (Figure 4B). As in LX2 cells, this decrease in survival was due to an increase in caspase activity that could be avoided by the addition of rIGF1 and rIGFBP5 (Figure 4C). Thus, IGFBP5 also increases the survival of human liver myofibroblasts by reducing apoptosis.

### Involvement of BCL2

**Figure 5:**
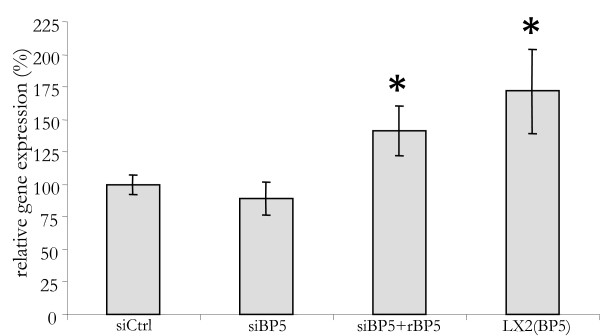
**IGFBP5 influences *BCL2 *expression**. LX2 cells were transfected with control small interfering RNA (siCtrl) or *IGFBP5 *siRNA (siBP5). Sixteen hours after transfection the medium was replaced by serum-free medium. Recombinant IGFBP5 was added at *t *= 24 h and 45 h, after replacing the medium, to siIGFBP5 transfected cells (siBP5+rBP5). After 48 h RNA was isolated from control (siCtrl), silenced (siBP5), silenced treated with rIGFBP5 (siBP5+rBP5) and LX2 cells overexpression IGFBP5 (LX2(BP5)) and used for quantitative polymerase chain reaction in order to determine *BCL2 *mRNA. The *BCL2 *mRNA levels were normalized to *36B4 *and given as a percentage of control cells.

In order to investigate the mechanism involved in the anti-apoptotic effect of IGFBP5 we studied its effect on the expression of several pro- and anti-apoptotic genes. The enhanced expression of *IGFBP5 *in transduced cells resulted in a significant induction of B-cell CLL/lymphoma 2 (*BCL2) *mRNA levels (Figure 5). Also, the addition of rIGFBP5 to *IGFBP5 *silenced LX2 cells resulted in a clear increase in *BCL2 *expression. The lack of effect of IGFBP5 silencing on *BCL2 *expression is unexpected and seems to suggest that, in presence of low levels of IGFBP5, other factors do determine (basal) *BCL2 *expression. *BCL2 *mRNA induction by high IGFBP5 levels seems to suggest that it could play a role in the anti-apoptotic effect of IGFBP5, but additional studies such as BCL2 silencing are needed in order to establish this possibility.

### Increased cell survival by IGFBP5 seems independent of IGF1

**Figure 6:**
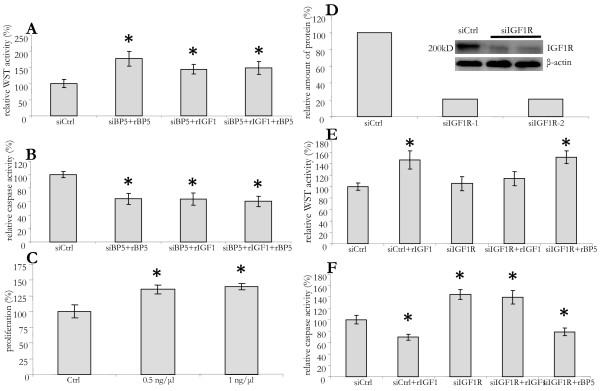
**Cell survival by IGFBP5 is not affected by IGF1 or mediated by IGF1R**. (A) LX2 cells were transfected with *IGFBP5 *small interfering RNA (siBP5). Sixteen hours after transfection the medium was replaced by serum-free medium for 48 h and then the viability was determined by performing a WST assay. Recombinant IGFBP5 (rBP5), IGF1 (rIGF1), or both (rIGF1+ rBP5), were added at 24 hand 45 h. (B) To cells treated as above, but cultured in 10% fetal calf serum, 0.5 μM gliotoxin was added at 45 h followed by a Caspase 3/7 assay 3 h later. (C) LX2 cells were cultured in serum-free medium for 48 h. rIGF1 was added in different concentrations at 24 h and 45 h, bromo-2'-deoxy-uridine (BrdU) was added at 32 h. Cells were lysed 16 h later (at 48 h) and assayed for BrdU incorporation. (D) LX2 cells were transfected with control (siCtrl) or siRNA for *IGF1R *(siIGF1R). Sixteen hours after transfection the medium was replaced by serum-free medium and cells were cultured for additional 48 h. The cells were then lysed and used for Western blot analysis in order to detect IGF1R. (E) Cells were transfected and cultured as in D. rIGF1 or rIGFBP5 (rBP5) was added at *t *= 24 h and 45 h after replacing medium. After 48 h a WST assay was performed. (F) 0.5 μM gliotoxin was added at 45 h, after replacing the medium. Caspase activity was measured 3 h later. All results are given as a percentage control cells.

IGFBP5 binds IGF1 with high affinity. Therefore, its protective role in LX2 cells and myofibroblasts could be due to modulation of the pro-survival effects of IGF1 signalling [20,35]. In order to investigate if IGFBP5 exerts its action via IGF1, we also determined the effect of IGF1 on LX2 cells viability. As with IGFBP5, the addition of IGF1 induced a 70% increase in survival of LX2 cells (Figure 6A), and lowering of apoptosis by 40% (Figure 6B). In order to investigate if IGF1 effect is modulated by IGFBP5, we studied the effect of the two factors added together. The response in viability and apoptosis were similar when IGF1 and IGFBP5 were added, alone or in combination (Figure 6A and 6B). However, in contrast to IGFBP5, IGF1 was able to enhance proliferation of LX2 cells for about 40% (Figure 6C), indicating that the two factors exert different effects on LX2 cells.

In order to establish a possible IGF1-independent effect of IGFBP5, we chose to silence IGF1R, the receptor that mediates IGF1 signalling. Transfection of LX2 cells with siRNA for IGF1R resulted in a 90% drop of IGF1R protein expression after 48 h (Figure 6D). This large decrease effectively inhibited the pro-survival effect of IGF1, but not that of IGFBP5 (Figure 6E). The possibility of IGF1-independent action of IGFBP5 was further confirmed by the addition of rIGFBP5 to *IGF1R*-silenced LX2 cells, which caused a decrease in caspase activity, even to the level below that in the control cells. The addition of IGF1, on the other hand, was not able to overcome the effect of IGF1R silencing and its effect on caspase activity was lacking (Figure 6F). Thus, in contrast to IGF1, the effect of IGFBP5 on LX2 cells survival is not mediated by the IGF1R, suggesting that the effect it exerts on partly activated stellate cells is not by modulation of IGF1 signalling.

### IGFBP5 and fibrotic markers

**Figure 7:**
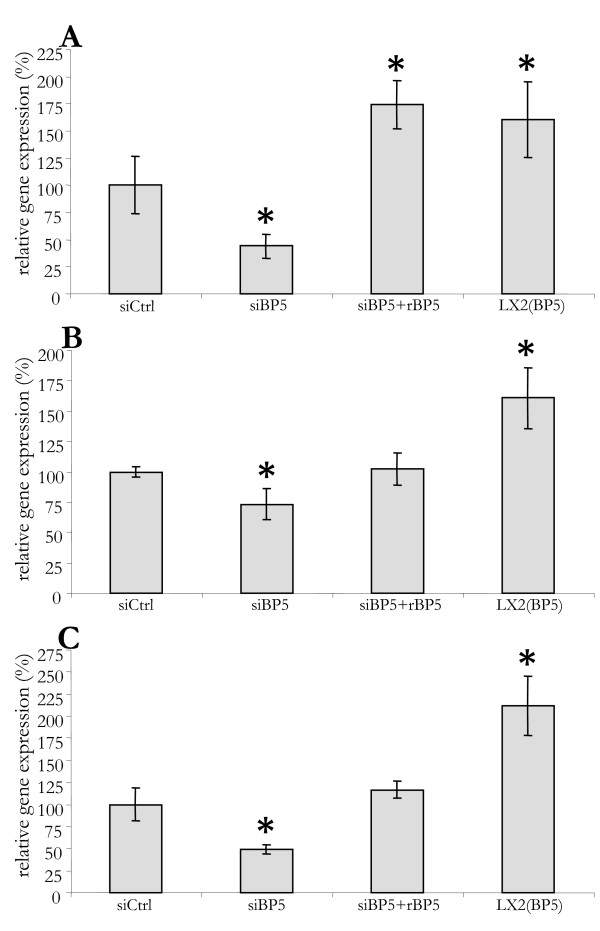
**IGFBP5 influences the expression of genes involved in fibrogenesis**. LX2 cells were transfected with small interfering control (siCtrl) or *IGFBP5 *siRNA (siBP5). Sixteen hours after transfection, medium was replaced by serum-free medium. Recombinant IGFBP5 (rIGFBP5) was added at 24 h and 45 h, after replacing the medium, to siIGFBP5 transfected cells (siBP5+rBP5). After 48 h of culturing on serum-free medium RNA was isolated from control, transfected and transfected treated with rIGFBP5. For comparison, RNA was isolated from LX2 cells overexpressing IGFBP5 cultured in serum-free medium (LX2(BP5)). Collagen 1α1 (A),*TIMP1 *(B) and *MMP1 *(C) mRNA levels were measured with quantitative polymerase chain reaction, normalized to *36B4 *and given as a percentage of control cells.

In tissues such as lung and skin, IGFBP5 was reported to enhance the expression of established pro-fibrotic markers, such as αSMA, collagen and fibronectin. As changed expression of these markers could further establish the (pro-fibrotic) role of IGFBP5 in liver fibrosis, we examined its influence on their expression in LX2 cells. As shown in Figure 7, overexpression of IGFBP5 induced the mRNA level of collagen, type I, alpha 1 (*COL1A1) *by 70%, tissue inhibitor of metalloproteinase (TIMP) metallopeptidase inhibitor 1 (*TIMP1) *by 70% and matrix metallopeptidase 1 (*MMP1) *by 100% in LX2 cells (Figure 7A-C). Silencing of *IGFBP5 *reduced the expression of these genes, which could be recuperated by the addition of recombinant IGFBP5. This indicates that IGFBP5 may not only enhance fibrosis by promoting the survival of activated HSC and myofibroblasts, but also by stimulating the expression of pro-fibrotic genes in these cells.

## Discussion

We have previously shown that IGFBP5 expression was strongly upregulated during HSC transdifferentiation *in vitro *and during development of liver fibrosis *in vivo *[9]. The aim of this study was to scrutinize the role of IGFBP5 in (partially) activated and transdifferentiated hepatic stellate cells.

IGFBP5 expression is increased in fibrotic lung, skin and liver [9,10,22,23]. The effects of IGFBP5 depend on cell type and tissue. For instance, in the process of mammary gland involution, IGFBP5 promotes apoptosis of epithelial cells *in vivo *and *in vitro *[36-39]. In contrast, an anti-apoptotic role of IGFBP5 has been reported, for instance, in myogenesis, in breast cancer cells grown *in vitro*, and in gingival epithelial cells [40-43].

In liver fibrosis, the level of apoptosis is reduced in activated HSC that play a pivotal role in the initiation and perpetuation of this pathological process [44]. Since IGFBP5 is induced in these cells upon activation and can promote survival, it may play a role in increasing the numbers of these activated cells seen in fibrotic liver. In order to study the effects of IGFBP5 *in vitro*, we used primary human myofibroblasts and LX2 cells, an established human model cell line for partially activated HSC [45-47]. The expression of *IGFBP5 *in LX2 cells was 30% higher than in quiescent normal HSC. For comparison, in cultured (that is, partially activated) HSC, *IGFBP5 *expression was doubled, while in hepatic myofibroblasts expression went up more than 200-fold. We used lentiviral transduction of LX2 cells to enhance the IGFBP5 expression to the levels seen in activated HSC. Upon serum depletion, both IGFBP5 over-expression and the addition of rIGFBP5 promoted the survival of LX2 cells by lowering the apoptosis. This suggests that the increased IGFBP5 expression could play a role in the reduction of apoptosis seen in activated HSC in the fibrotic liver. In accordance, lowered *IGFBP5 *expression by RNA silencing decreased the viability of LX2 cells. *IGFBP5 *silencing also decreased survival in human primary liver myofibroblasts. In both cell types this was due to increased apoptosis, demonstrating that IGFBP5 functions as an anti-apoptotic, pro-survival factor in these two pro-fibrotic cell types. Two recent studies reported a high expression of IGFBP5 in hepatocellular carcinoma (HCC) and intra-hepatic cholangiocarcinoma (CC), suggesting that it has a similar role *in vivo *by promoting the survival of cancer cells [48,49]. We demonstrated that IGFBP5 expression strongly increased during development of liver fibrosis in Mdr2^-/- ^mice [9]. This model of liver fibrosis not only spontaneously develops portal fibrosis, but is also prone to HCC formation [50,51]. The presence of IGFBP5 in this animal model and in patients with HCC or CC suggests that, in addition to a role in the pathogenesis of liver fibrosis, IGFBP5 may also contribute to tumour formation. Lowering IGFBP5 expression may, therefore, not only impair the development of liver fibrosis but also reduce the risk of tumour formation.

The role of IGFBP5 in IGF1 signalling is well established. IGFBP5 binds IGF1 with high affinity and protects it from rapid degradation [20] but, at the same time, prevents induction of the IGF1R and thereby inhibits pro-survival signalling by IGF1. The observed effects of IGFBP5 may therefore be due to its role in IGF1 signalling - that is to its disturbance of the IGF1 axis observed in liver fibrosis [19]. Our *in vitro *studies show, however, that, in contrast to IGFBP5, IGF1 increases proliferation of LX2 cells. Moreover, no effect of IGF1 on IGFBP5 action, nor of IGFBP5 on IGF1 signalling, was detected. Therefore, it seems that the two factors exert their effect on LX2 cells via different routes. In line with this, silencing of IGF1R resulted in loss of the pro-survival effects of IGF1 but did not interfere with the pro-survival effects of IGFBP5. This strongly supports the notion that IGFBP5 promotes survival of activated HSC by lowering apoptosis in an IGF1-independent manner. Several mechanisms, such as activation of TGFβ1, modulation of the coagulation cascade and integrin activation may be involved in the IGF1-independent effect of IGFBP5 [52-54]. In addition, the existence of an IGFBP5 receptor has been proposed to explain the IGF1-independent actions of IGFBP5 [53,54].

The finding that IGFBP5 promoted survival without inducing proliferation suggests that it may induce cellular senescence. Overexpression of IGFBP5 is shown to induce senescence in HUVEC cells by induction of the tumour suppressor p53 [26]. The induction of senescence plays a promoting role in the development of pulmonary fibrosis [24]. On the other hand, senescence in activated HSC was associated with impediment of liver fibrosis [55], leaving the role of IGFBP5 in the development of senescence in liver fibrogenesis opened for further inquiries. Additionally, adenovirally mediated overexpression of IGFBP5 induced the expression pro-fibrotic genes and ECM deposition in lung and skin [22,23]. We showed that IGFBP5 also enhanced the expression of genes directly involved in liver fibrogenesis such as *collagen1α1, TIMP1 *and *MMP1 *in LX2 cells. Its effect on these pro-fibrotic genes in a model for activated HSC indicates that IGFBP5 may also have a direct effect on the ECM deposition in liver fibrosis. Increase of both *TIMP1 *and *MMP1 *expression, and thus exertion of both fibrotic and matrix-degrading effects, could be seen in the light of a necessity to establish a balance between matrix deposition and restructuring during the process of fibrogenesis. However, since our data only demonstrate changes on the mRNA level, it seems possible that the changes in the expression of these opposing factors may not have occurred on the protein level due to posttranscriptional and posttranslational modifications.

In conclusion, our data show that IGFBP5 improves the survival of (partially) activated HSC and liver myofibroblasts by lowering the level of apoptosis via an IGF1-independent mechanism. In addition, IGFBP5 increases the expression of genes involved in ECM deposition. Accordingly, lowering expression of this apparently pro-fibrotic factor may impair the progression of liver fibrosis.

## Abbreviations

BCL2: B-cell CLL/lymphoma 2; BrdU: bromo-2'-deoxy-uridine; CC: cholangiocarcinoma; ECM: extracellular matrix; FCS: fetal calf serum; HCC: hepatocellular carcinoma; HSC: hepatic stellate cells; IGF1: insulin-like growth factor 1; IGF1R: insulin-like growth factor 1 receptor; IGFBP5: insulin-like growth factor binding protein 5; MMP1: matrix metallopeptidase 1; PARP: poly (ADP-ribose) polymerase; PGK: phosphoglycerate; qPCR: quantitative polymerase chain reaction; rIGFBP5: human recombinant insulin-like growth factor binding protein 5; siRNA: small interfering RNA; TGFβ1: transforming growth factor, beta 1; TIMP1: tissue inhibitor of metallopeptidase inhibitor 1.

## Competing interests

The authors declare that they have no competing interests.

## Authors' contributions

AS carried out the experimental part of the study and prepared the manuscript. MS took part in the experiments and writing. WB provided an insight into the SAGE database. ROE and PB supervised the study.
